# Public Expenditure and Green Total Factor Productivity: Evidence from Chinese Prefecture-Level Cities

**DOI:** 10.3390/ijerph19095755

**Published:** 2022-05-09

**Authors:** Weixiang Zhao, Yankun Xu

**Affiliations:** 1 School of Public Finance and Taxation, Zhongnan University of Economics and Law, Wuhan 430074, China; zhaoweixiang@stu.zuel.edu.cn; 2 School of Economics, South-Central Minzu University, Wuhan 430074, China

**Keywords:** public finance, expenditure size and composition, green total factor productivity, China

## Abstract

Whilst effective public expenditure policies are essential for transforming the traditional factor-driven economy into a green and innovation-driven economy, the impacts of public expenditure’s size and composition on green economic development have not been comprehensively investigated. This paper attempts to fill this research gap. Based on the data of Chinese prefecture-level cities from 2010 to 2018, we first measure green total factor productivity (GTFP), the proxy variable for green development, and briefly analyze its spatial-temporal trends. Then, using the dynamic panel models, dynamic panel mediation models, and dynamic panel threshold models, we evaluate how public expenditure affects GTFP. The main findings are fourfold: (1) there is a significant inverted U-shaped relationship between the expenditure size and GTFP. (2) The expansion of social expenditures and science and technology (S&T) and environmental protection expenditures play an important role in stimulating green growth, while economic expenditures and administrative expenditures have adverse effects. (3) Public expenditure mainly promotes green development through four channels: human capital accumulation, technological innovation, environmental quality improvement, and labor productivity increase. (4) The expenditure composition influences the turning point of the inverted U-shaped relationship. Based on these findings, we propose some targeted policy suggestions to promote green development.

## 1. Introduction

Pollutant emissions from industrial and rapidly industrializing economies directly lead to worldwide environmental issues such as pollution and climate change, posing severe threats to public health [1,2]. In order to reconcile the conflicting relationship between human economic activities and the environment, more and more countries have been attempting to pursue green economic development, which deviates from the traditional extensive growth strategy and aims at improving welfare and social equity while significantly reducing environmental risk and ecological scarcity [3,4]. The realization of green development requires the participation of micro-market players and social subjects, such as enterprises increasing technological innovation and residents greening their consumption and investments [5,6]. However, considering that green behavior has obvious positive externalities, various participants’ green behavior may not show incentive compatibility, which may lower the efficiency and the subjects’ willingness to promote the transition towards a greener economy. In this case, relying on the guidance, regulation, and financial compensation of public expenditures financed by taxation to achieve the goal of overall green development transformation is reasonable, legitimate, and inevitable. 

The impacts of public expenditures on green development are multidimensional and double-edged. In the economic growth model, public expenditures have significant positive externalities and knowledge-spillover effects. Expenditures on education and healthcare help to promote economic growth from being physical capital-driven to human capital and technology-driven. Environmental protection expenditures help to internalize the environmental externalities associated with production activities and provide greater incentives for green behavior. Finally, expenditures on science and technology (S&T) promote innovation, particularly green innovation. However, the size and composition of public expenditure can also have negative consequences for green development. Excessive government expenditures will crowd out effective social resources, which is not conducive to the overall efficiency of society. For example, a large scale of economic spending may lead to excessive government intervention in the private sector and weaken the fundamental driving force of economic development. Too-high administrative expenditures may indicate that the government is inefficient and will reduce economic efficiency. Therefore, an appropriate size and reasonable composition of government expenditure are of great significance to promoting green transformation and development.

To date, a few studies have focused on the impacts of fiscal behavior, especially public expenditure, on green growth. Lopez et al. [7] were the first to analyze the environmental effects of public expenditure. Their theoretical analysis found that increasing the size of public expenditure was conducive to a reduction in air and water pollutants, while this positive impact turns neutral when the proportion of public expenditure to private expenditure remains unchanged. Halkos and Paizanos [8] used a dataset containing 77 countries or regions to study the impacts of government spending on air pollution and found that government expenditures had a significant negative impact on dioxide emissions per capita, especially in low-income countries. Hua et al. [9] constructed an Optimal Control model and described a negative relationship between education expenditure, S&T expenditure, and air pollution, which showed a decreasing trend from coastal to inland areas. Using the panel data of Chinese cities, Lin and Zhu [10] applied a non-radial distance function in constructing a green economic growth index. They conducted a system GMM estimation and derived that the education expenditure and R&D expenditure can promote green economic growth. Postula and Radecka-Moroz [11] took the European Union as an example to analyze the role of fiscal tools in environmental protection and found that public expenditure only had a long-term effect. 

Nevertheless, these existing studies are relatively limited. They mainly support a linear relationship between public spending size and environmental pollution or green development, while the crowing out effect of excessive government spending is little explored. From the perspective of the composition of public expenditure, environmental economists have analyzed the environmental effects of education and S&T expenditures, and little attention has been paid to the impacts of economic expenditures, environmental expenditures, and administrative expenditures. In addition, almost all current literature focuses on the impact of fiscal spending on pollution emissions, and the research on the impact on green development performance remains insufficient. Given that public expenditures have multiple environmental and economic effects, the question of how to promote the coordination of environment and economic development has become a fundamental goal of green transformation. While considering the environmental and economic development goals, this paper tries to fill the aforementioned literature gaps by comprehensively analyzing the impacts of public expenditure size and composition on green development performance using city-level panel data from China.

The paper most similar to ours is Lin and Zhu [10], which focused on the impact of education expenditure and R&D expenditure on the green economic growth index. By comparison, our work mainly expands on three aspects. First, we elucidate the stages of China’s green development transition and public-expenditure policies. Afterward, we reveal that the impact of public expenditures on green development is mainly conducted in two dimensions, which are the expenditure size and structure. Second, we measure green total factor productivity (GTFP) as a proxy variable for green development using a global non-angle, non-radial DEA-SBM model combined with the GML (Global Malmquist–Luenberger) index. We briefly analyze the trends, structure, and regional distribution of GTFP. Third, we construct dynamic panel models, dynamic panel mediation models, and dynamic threshold panel models to examine the impact of public expenditure on GTFP in detail. Specifically, we find that the size of public expenditure and GTFP show a clear inverted U-shaped relationship, and there are significant differences in the impact of various categories of public expenditure on GTFP, namely, economic expenditures, social expenditures, administrative expenditures, S&T, and environmental protection expenditures. Among them, social expenditures and expenditures on S&T and environmental protection significantly promote green transformation and development. Our results also show that the impact of public expenditures on GTFP is mainly transmitted through four channels, namely, human capital accumulation, science and technology innovation, environmental quality improvement, and labor productivity increase. Social expenditures positively affect GTFP by promoting human capital accumulation, technological innovation, and increasing labor productivity. S&T and environmental protection spending positively affect GTFP through all four channels. Last but not least, we find that changes in the spending composition affect the inverted U-shaped relationship between expenditure size and GTFP. When the proportion of social expenditures and S&T and environmental protection expenditures and administrative expenditures reach their respective threshold values, the value of the turning point of the inverted U-shaped relationship between public expenditure size and GTFP will be increased, creating more room for the expansion of public spending to promote green development.

This paper contributes to the literature in the following ways. We comprehensively study the impacts of public expenditures on green development. The existing research mainly focuses on how pollution emissions, energy consumption, and ecological conservation can be affected by public expenditures. Only a small amount of attention has been paid to the impact of public spending on green development. This paper fills this literature gap by explaining and evaluating the impacts of the size and composition of public expenditure on GTFP. Additionally, our research reveals and verifies the underlying mechanisms of public expenditure’s impacts from an empirical perspective. In particular, considering the impacts of different types of public expenditures on green development performance and the impacts of different types of public expenditures on different mediation variables helps in more accurately understanding the complex relationship between public expenditure and green development. Finally, our research has important implications for developing countries, especially for transitional countries in the process of green transformation and development, in the design of public expenditure policies. Public expenditure policy should take into account not only appropriate size but also structural optimization, and the policymaker should accurately recognize the stages and driving forces of the green development transformation and design policies with these in mind.

The rest of this study is organized as follows. Section 2 reviews the background and characteristics of public expenditure policies and green development transformation in China and analyzes the mechanisms of public expenditures’ impact on green development along the dimensions of size and composition. Section 3 introduces a global non-angle and non-radial DEA-SBM model combined with the GML index to measure GTFP and analyze the distribution of GTFP from spatial and temporal perspectives. Section 4, mainly for the preparation of empirical analysis, provides the details of empirical models, methods, and data used in this paper. Section 5 includes an empirical analysis examining the impact of public expenditure on GTFP and its mechanisms and interpreting the results. Section 6 concludes with some targeted policy suggestions.

## 2. Institutional Background and Theoretical Mechanism Analysis

### 2.1. The Green Development Strategy and Public Expenditure Policies in China

China’s green development strategy, which is the core content of its “national strategy of ecological civilisation”, was developed in the context of attempting to change China’s extensive economic growth since the reform and opening up. Since it became a national strategy in 2012, China has issued a number of national action plans regarding air, water, soil, solid waste, ecological resources, etc., with the aim of reversing the current trend of the rapid deterioration of ecological and environmental quality. At the same time, ecological and environmental considerations have also begun to be integrated into other economic and social policies, including fiscal policy, monetary policy, industrial policy, land policy, judicial policy, and social security policy. This rapid “greening” of China’s public policies provides a useful context for us to study the progress of green development and its driving mechanisms.

The fiscal system has a special status and importance in China. The establishment of a modern fiscal system has been clearly established as the foundation, and an important pillar, of the country’s national governance capacity and the modernization of its systems. Fiscal policy has also been widely used in ecological and environmental governance. In 2007, environmental expenditure became an independent expenditure item in China’s public budget, alongside education, medical care, social security, and other listed expenditure items. From the government budget perspective, China’s public expenditure includes four main types: general public budget expenditure, budgetary expenditures of government-managed funds, social security budget expenditure, and state-owned capital budget expenditure. While the latter three types of expenditure are mainly arranged for specific fields and purposes, the general public budget expenditure has the characteristics of universality, extensiveness, and transparency. In this paper, therefore, we mainly focus on China’s general public budget expenditure. 

In 2007, China carried out a reform regarding the classification of budgetary revenues and expenditures, which is still in effect today. Public expenditure is classified into 18 types of specific components. Based on the economic property of each component, they can be further divided into several categories [12,13]. Referring to Jia et al. [14] and Wu et al. [15], we classify public expenditures into four types in this paper, namely, social expenditures, economic expenditures, science and technology (S&T) and environmental protection expenditures, and administrative expenditures. Social expenditures include education expenditure, cultural, sports and media expenditure, social security and employment expenditure, medical and health and family planning expenditure, urban and rural community expenditure, and housing security expenditure. Economic expenditures comprise expenditures on agriculture, forestry, water, and transportation. S&T and environmental protection expenditures are composed of scientific and technological innovation expenditure and energy conservation and environmental protection expenditure, while administrative expenditures consist of public service expenditure and public security expenditure. Table 1 shows the changes in the size and structure of China’s local government fiscal spending from 2010 to 2018. As a whole, the largest expenditure category for local governments in China was social expenditures, which accounted for 54.88% of total expenditure in 2018. In the same year, the proportions of economic expenditures, administrative expenditures, and environmental protection and S&T expenditures in total public spending were in descending order. During the sample period, social expenditures and S&T and environmental protection expenditures showed a steady upward trend, while administrative expenditures presented a downward trend. Economic expenditures have a tendency of first rising and then falling within a given period.

### 2.2. Mechanisms of the Impact of Public Expenditure on Green Development

Public expenditure mainly affects green development by influencing the ecological and environmental quality and economic growth [10]. Public expenditure is largely financed through taxes, which per se are an important channel to internalize negative externalities and, to a certain extent, consider the cost of energy and environmental and ecological consumption. Neutral tax policies help to promote economic growth and productivity. Public expenditure is mainly used to supply public goods, which have strong positive externalities. By providing public goods, public spending can help to increase the marginal productivity of various production factors that promote economic growth. The increase in productivity itself implies an increase in the efficiency of resources and energy consumption and a decline in pollutant emissions. Moreover, expenditure can help to compensate, guide, and support actions with strong externalities, such as clean energy, pollution-reduction technologies, and ecological environment governance. 

Theoretically speaking, a certain size of public expenditure can act on green development through taxation and public products to promote green transformation and development. However, excessive public expenditure may inhibit green growth, mainly in the form of a crowding-out effect. Disproportionate allocation of resources to the public sector can crowd out factor resources required by the private sector’s production activities, which may raise the private sector’s production costs, such as credit costs and human costs. This, in turn, is not conducive to the improvement of production efficiency. Moreover, in China, although public resources can be partially transferred to the private sector through subsidies and tax incentives, these resources are usually disproportionately allocated to state-owned enterprises. The fund-leakage phenomenon for the private sector is evident, particularly affecting green innovation behaviors that rely more on external subsidies and tax incentives [16]. Meanwhile, Baumol’s cost disease exists in the public sector. Excessive resource allocation to the public sector is not conducive to the improvement of public sector efficiency, and low public efficiency can further drag down the efficiency of the private sector [17]. Therefore, there may be an inverted U-shaped relationship between public expenditure and green development.

In this paper, taking differences in the types of public expenditure into account, we further divide the total expenditure into four categories, namely, social expenditures, economic expenditures, S&T and environmental protection expenditures, and administrative expenditures, which can cover most expenditure types [10,14]. Considering the different purposes and economic properties of each category of expenditure, they may have heterogeneous effects on green development. Social expenditures, mainly used in the areas of education, healthcare, health, and social security, are an important way of accumulating and enhancing human capital, especially for developing countries and countries in transition. Public sector investment in social spending can compensate for the negative impact of insufficient private investment. Human capital is also considered to be an essential way to induce innovation and an important channel for increasing labor productivity and total factor productivity [9,10]. Along with the increase in the level of human capital, the demand for clean products will further increase, which will force the production of polluting products to be reduced and improve the overall efficiency of clean production. Meanwhile, an increase in the level of human capital will also help to improve the overall resource allocation and management capabilities of society. Overall, social spending with human capital improvement as the core could contribute to green development. 

Economic spending is mainly directed at improving the efficiency of resource allocation and maintaining economic stability in areas of market failure. However, since market failure is not a necessary and sufficient condition for government intervention, the government also faces the risk of failure under market conditions. In the early stage of economic development, a certain amount of economic expenditures can help compensate for the lack of private investment. For the middle and late stages of economic development, economic spending tends to have crowding-out effects on private investment and consumption behavior, raising the cost of private economic behavior and inhibiting economic efficiency [18]. Meanwhile, compared with non-economic expenditures, economic expenditures mainly belong to physical capital investment and industrial investment. Investment in capital-intensive industries often causes considerable energy consumption and pollution emission. Therefore, the increase in economic expenditures may hinder green development.

S&T and environmental protection expenditures mainly involve public expenditures for technological innovation, energy conservation, and environmental protection activities. S&T expenditure can help promote R&D and technology spillover effects, encourage cleaner production behaviors, and improve total factor productivity [19,20]. Most environmental protection expenditure is used to compensate and subsidize ecological, environmental governance, and resource and energy conservation and provide incentive and guidance for positive external private-sector environmental behaviors. Moreover, environmental protection expenditures are primarily oriented toward green innovations, energy-saving technologies, and ecological protection and have direct green production attributes [21,22,23,24,25,26]. In brief, S&T and environmental protection expenditure can boost the green development of the economy. 

Administrative expenditures are the basis for the regular operation of the government. They are purely expendable and do not directly contribute to economic growth and ecology. Given the limited public funds, excessive administrative expenditure is detrimental to overall economic growth and green development [7,14]. Because administrative expenditures are mainly used for staff salaries and benefits and the functioning of the state apparatus, excessive administrative spending is an essential indication of inefficient and costly government operations, which is detrimental to overall green transformation and development.

This section detailedly analyzes the theoretical impacts of the size and composition of public expenditure on green development. It is relatively evident that human capital accumulation, scientific and technological innovation, environmental quality, and labor productivity are the main channels through which public expenditure affects green development. Thus, we regard them as mediation variables in our empirical analysis to analyze the mechanisms of the green effect of public expenditure. In addition, there may be an inverted U-shaped relationship between public expenditure and green development. The curve’s turning point may indicate room for the expansion of public expenditure to enhance green development. We will verify the existence of this possible inverted U-shaped relationship in an empirical analysis and examine whether changes in the proportion of each category of public expenditure alter the impact of public spending’s expansion on green development. 

## 3. The Construction of GTFP and Its Distribution

### 3.1. The Calculation Method

After years of rapid development, China’s economic dynamism has been greatly improved. However, China’s extensive growth, characterized by the high consumption of resources and energy and high pollutant emissions, has brought side effects, namely, “unbalanced, uncoordinated and unsustainable development”. Therefore, increasing green total factor productivity (GTFP), which is an essential indicator to measure the level of green and high-quality development [27], has become a fundamental way to promote green economic transformation and achieve environmentally friendly and sustainable development.

In this paper, we treated city-level GTFP as the proxy variable to measure China’s green development. We used the non-radial, non-angle slack-based measure (SBM) proposed by Tone [28], which is able to cover non-desired output variables in the production process and supports slack improvement, to accurately evaluate urban environmental efficiency. We took the prefecture-level cities in China as decision-making units *(*$DMU_{i}^{t},\;i=1,\ldots ,I;t=1,\ldots ,T$*)* to construct production frontiers. The number of input factors *x* for each decision unit is *n*, including labor (L), capital stock (K), water supply (W), and electricity consumption (E) (it is worth noting that most scholars choose “coal consumption” or “oil consumption” to measure energy inputs in national and provincial level studies. However, data on coal and oil at the city level are currently not available, and data on gas and LPG are missing, so this paper adopts social electricity consumption as a proxy for urban energy input [10,29,30]). The decision unit uses input factors to produce *m* desired outputs *y*, including gross domestic product (GDP) and greening coverage (G) of each region, and *j* non-desired outputs *b*, including industrial wastewater (IW), sulfur dioxide emission (SDE), and soot emission (SE). The data needed to measure each variable are obtained from the China Urban Statistical Yearbook, and the definitions are shown in Table 2. In summary, the production possibility set can be described as:

(1) $$P^{t}\left(x^{t},y^{t},b^{t}\right)=\left\{\left(y^{t},b^{t}\right)丨x^{t}\;can\;produce\left(y^{t},b^{t}\right)\right\}$$

In Equation (1), $P^{t}\left(x^{t},y^{t},b^{t}\right)$ contains the input–output conditions of all prefecture-level cities in period *t*. Combining Equation (1) with the directional distance function, we can calculate the GTFP change index for each prefecture-level city at each period. However, the results obtained using this classical algorithm are not cyclic and have the drawback of being unsolvable by linear programming [31]. Therefore, we calculated the GML (Global Malmquist–Luenberger) index constructed by Oh [32] that simultaneously scales the production frontier for all periods to address the above issues. The global production possibility set can be expressed as: $PG=P^{1}\cup P^{2}\cup \ldots \cup P^{T}$. According to Li [33], the SBM-DDF model with constant payoffs to scale when applying the global production possibility set is shown in the following equation:

$$\hat{P}G_{i}^{t}\left(X_{i}^{t},Y_{i}^{t},B_{i}^{t}\right)=\max[w_{n}^{x}\overset{N}{\underset{n=1}{\sum }}\frac{{\hat{\beta }}_{n,i}^{X-t}}{N}+w_{m}^{y}\overset{M}{\underset{m=1}{\sum }}\frac{{\hat{\beta }}_{M,i}^{Y-t}}{M}+w_{j}^{b}\overset{J}{\underset{j=1}{\sum }}\frac{{\hat{\beta }}_{j,i}^{B-t}}{J}]$$

$$S.t\sum_{t=1}^{T}\sum_{i=1}^{I}\theta_{i}^{t}X_{in}^{t}\leq \left(1-{\hat{\beta }}_{in}^{X-t}\right)X_{in}^{t},\;\forall n;$$

(2) $$\sum_{t=1}^{T}\sum_{i=1}^{I}\theta_{i}^{t}Y_{im}^{t}\geq \left(1-{\hat{\beta }}_{im}^{Y-t}\right)Y_{im}^{t},\;\forall m;$$

$$\sum_{t=1}^{T}\sum_{i=1}^{I}\theta_{i}^{t}B_{ij}^{t}=\left(1-{\hat{\beta }}_{ij}^{B-t}\right)B_{ij}^{t},\;\forall j;\;\theta_{i}^{t}\geq 0,\;i=1,\ldots ,I;t=1,\ldots ,T;n=1,\ldots ,\;N;m=1,\ldots ,\;M;\;j=1,\ldots ,\;J$$

where the constant returns to scale assumption removes the differences in results from input- and output-oriented calculation of the GML index; $w_{n}^{x},\;w_{m}^{y},\;w_{j}^{b}$ denote the weight vectors of inputs, desired outputs, and undesired outputs, respectively. Under the general assumption that the inputs and outputs are equally important, the weights for them are $\frac{N}{N+M+J},\;\frac{M}{N+M+J},\;\frac{J}{N+M+J}$, respectively. The scale vector ${\hat{\beta }}_{i}^{t}$ is represented using the optimal solution of ${\hat{\beta }}_{i}^{t}=\left({\hat{\beta }}_{n,i}^{X-t},\;{\hat{\beta }}_{M,i}^{Y-t},\;{\hat{\beta }}_{j,i}^{B-t}\right)$, which can ensure that both the input and undesired output scale reduction and the desired output scale expansion of the decision unit are maximized. $\theta_{i}^{t}$ is the intensity variable of $DMU_{i}$ at *t*, which is a constant vector of I×1. Combining all the constraints above, Equation (2) is able to find the optimal state where the minimum amount of resources is invested, and the maximum desired output and the minimum undesired output can be achieved, i.e., the global production frontier. We refer to Oh [32] and express the GML index under the assumption of constant returns to scale as follows:

(3) $$GML^{t,t+1}\left(x^{t+1},y^{t+1},b^{t+1},x^{t},y^{t},c^{t}\right)=\frac{1-PG^{t+1}\left(x^{t+1},y^{t+1},b^{t+1}丨C\right)}{1-PG^{t}\left(x^{t},y^{t},b^{t}丨C\right)}$$

A greater than one $GML^{t,t+1}$ index of a city means an increase in GTFP for this city from *t* to *t +* 1 and vice versa. The GML index can be further decomposed as follows:

(4) $$GML^{t,t+1}=\left[\frac{1-PG^{t+1}\left(x^{t+1},y^{t+1},b^{t+1}丨C\right)}{1-P^{t+1}\left(x^{t+1},y^{t+1},b^{t+1}丨C\right)}/\frac{1-PG^{t}\left(x^{t},y^{t},b^{t}丨C\right)}{1-P^{t}\left(x^{t},y^{t},b^{t}丨C\right)}\right]\;\;\times \left[\frac{1-P^{t+1}\left(x^{t+1},y^{t+1},b^{t+1}丨V\right)}{1-P^{t}\left(x^{t},y^{t},b^{t}丨V\right)}\right]\times \left[\frac{1-P^{t+1}\left(x^{t+1},y^{t+1},b^{t+1}丨C\right)}{1-P^{t+1}\left(x^{t+1},y^{t+1},b^{t+1}丨V\right)}/\frac{1-P^{t}\left(x^{t},y^{t},b^{t}丨C\right)}{1-P^{t}\left(x^{t},y^{t},b^{t}丨V\right)}\right]\;=GTPCH^{t,t+1}\times GPECH^{t,t+1}\times GSECH^{t,t+1}$$

where *C* denotes constant returns to scale, and *V* denotes variable returns to scale. $GTPCH^{t,t+1},\;GPECH^{t,t+1},\;\;and\;GSECH^{t,t+1}$ represents the technological progress index, pure technical efficiency index, and scale efficiency index, respectively. The three indices greater than, equal to, or less than 1 indicate that the level of technology, level of pure technical efficiency, and level of scale efficiency of a prefecture-level city increase, remain unchanged, and decrease from period *t* to period *t +* 1, respectively.

### 3.2. The spatial-Temporal Patterns of GTFP and Its Composition

#### 3.2.1. Overall Analysis

O’Donnell [34] points out that the GML index is not the total factor of productivity. Drawing on Li and Wu [35], we calculated green total factor productivity (GTFP) and technological progress (GTECH), pure technical efficiency (PGEFFCH), and scale efficiency (SGEFFCH) for each prefecture-level city of each period using the following formulas:

(5) $$GTFP^{t}=1\times GML^{1}\times \ldots \times GML^{t}\;GTECH^{t}=1\times GTPCH^{1}\times \ldots \times GTPCH^{t}\;PGEFFCH^{t}=1\times \ldots \times GPECH^{t}\;SGEFFCH^{t}=1\times \ldots \times GSECH^{t}\;$$

We present the main results in Table 3 and Table 4. We can see from Table 3 that the average values of GTFP in China increased from 1.070 in 2010 to 1.147 in 2018, indicating that China in general achieved green development during the sample period selected for this paper. Specifically, green total factor productivity at the prefecture-level city level in China showed a trend of oscillating growth, consistent with the results in Chen et al. [36]. Moreover, we present the calculation results of GTFP from 2010, 2015, and 2018 in the form of topographic maps to intuitively reflect the regional differences and dynamic changes of the GTFP (these three periods are selected because 2010 and 2018 are the starting and ending years of our sample period, and 2015 is the last year that the overall GTFP incurs a decrease. Therefore, we believe that choosing these three periods can help us better understand the dynamics of China’s green development).

**Figure 1:**
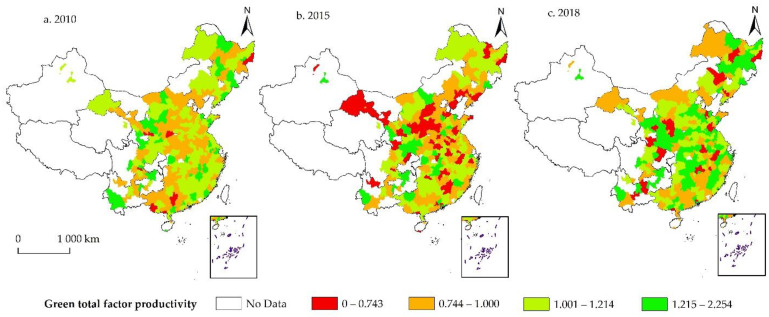
Spatial-temporal pattern of GTFP in China during 2010–2018.

Comparing the three maps (as shown in Figure 1), we can see that the number of red and orange areas on the map increased from 2010 to 2015, reflecting a reduction in the level of green total factor productivity in China’s cities, which is consistent with Xia and Xu [37]. This is due to the “after-effects” of China’s four-trillion investment plan in 2008 to stabilize the economy in response to the US’s subprime mortgage crisis that became gradually apparent between 2010 and 2015. The investment plan was effective in stimulating economic growth, but the huge amount of capital generated was largely invested in heavy industries such as steel and coal, and mining, which led to an increase in problems such as overcapacity and environmental pollution, seriously affecting the quality of China’s economic development.

The 2018 map shows a significant increase in green areas compared to 2010 and 2015 and a considerable decrease in orange and red areas, implying an improvement in China’s economic development quality (specifically, 167 cities saw their GTFP improve in 2018 compared to 2010, while 227 cities saw their GTFP improve compared to 2015). China’s GTFP has gradually improved since 2016, mainly due to the country’s focus on building an ecological civilization and combating pollution since the 18th National Congress and the 13th Five-Year Plan. Furthermore, Chinese central and local governments continued to encourage economic restructuring and industrial upgrading, contributing to high-quality economic growth and improved resource efficiency.

We also present how the components of GTFP changed on average at the national level over time in Table 3. The table shows that Chinese cities as a whole have witnessed a downward trend in their GTECH, indicating that they may have suffered a significant decline in the level of technology (technological progress indicates an outward shift in the global production frontier. The production frontier is shifted outwards when and only when the input–output ratio of the city on the production frontier decreases, which indicates an improvement in technology and vice versa). Technological regression seems to be contrary to reality [38,39]. However, regions in China generally faced increasing input–output ratios since the scale of factors of production was expanding faster than the scale of the desired output. Such changes would undoubtedly lead to a year-on-year inversion of the production frontier and consequently to a technological regression. This phenomenon does not imply a real decline in the level of production technology.

The results in Table 3 also indicate that improvements in pure technical efficiency (PGEFFCG) and scale efficiency (SGEFFCH) were the main engines of green and high-quality economic growth in China over the sample period. The increase in pure technical efficiency implies a significant improvement in the ability of cities to allocate productive resources appropriately and use them efficiently. The improvement in scale efficiency suggests that cities focus on exploiting the economies of scale brought about by the increased scale of production resources. The results of this paper also indicate that pure technical efficiency played a more significant role in China’s green development process than scale efficiency.

#### 3.2.2. Regional analysis

The level of regional green development varies from region to region in China due to differences in regional development strategies (for example, China implemented the “Eastern Coastal Opening” strategy in 1978, the “Western Development” strategy in 2000, the “Revitalisation of the Old Northeast Industrial Base” strategy in 2003, and the “Rise of Central China” strategy in 2006) and resource-endowment conditions. We divide China into eastern, northeastern, central, and western regions (the eastern region comprises ten provinces, including Beijing, Tianjin, Hebei, Shanghai, Jiangsu, Zhejiang, Fujian, Shandong, Guangdong, and Hainan province. The central region includes Shanxi, Anhui, Jiangxi, Henan, Hubei, and Hunan. The western region includes Inner Mongolia, Guangxi, Chongqing, Sichuan, Guizhou, Yunnan, Shanxi, Gansu, Qinghai, Ningxia, and Xinjiang. The northeast region includes Liaoning, Jilin, and Heilongjiang) in order to analyze the GTFP and its composition in each region to reflect these differences.

Table 4 shows that GTFP in all four major regions of China improved over the sample period. At the time of 2018, the central region had the highest level of green development, followed by the northeastern, eastern, and western regions. As a result of actively improving the coordination among production factors and learning management techniques, both pure technical efficiency and scale efficiency in the Northeast region increased highly over time, leading to improved green development in the Northeast region. However, the rate of technological progress in Northeast China was the lowest among the four major regions during the sample period. This indicates that its development strategy, which focused on labor- and pollution-intensive industries, only yielded relatively low positive output and high undesired output after investing in production resources. Similarly, China’s central and western regions also achieved green growth over the sample period and showed relatively significant increases in pure technical efficiency and technical efficiency at scale. Finally, we find that the green growth pattern in the eastern region differs from the other areas. It relied primarily on improvements in technology levels to drive green and high-quality economic development. Local governments in the eastern region actively encouraged enterprises to develop green production technologies and pollutant treatment and reuse technologies to achieve green, high-quality production [40]. In addition, the abundant human capital in the eastern region helped to promote the further transformation of the industrial structure towards a more specialized, advanced, and high-quality one, which also indirectly reduced energy consumption per unit of GDP. As Färe et al. [41] stated, improving production technologies and pollution treatment technologies can reduce pollution emissions and energy consumption and ultimately increase GTFP.

## 4. Econometric Methodology

### 4.1. Dynamic Panel Model

#### 4.1.1. The Impacts of Fiscal Expenditure Size on GTFP

Improving GTFP is essential in achieving green growth. Changes in the size of fiscal spending, which represents an important method of government intervention in the economy, will inevitably have an impact on GTFP. Therefore, we use the GTFP of Chinese cities calculated by Equations (4) and (5) as the dependent variables and establish a regression model to explore the effect of the size of public expenditure on green, high-quality growth. The specific model is as follows:

(6) $$GTFP_{it}=\alpha_{0}+\alpha_{1}wperexp_{it}+\alpha_{2}wperexp2_{it}+\theta X_{it}+\mu_{i}+\tau_{t}+\varepsilon_{it}$$

where $wperexp_{i,t}$ denotes the size of fiscal expenditure. Drawing on the existing studies, this paper selects per capita fiscal expenditure as a measure of expenditure size [29,42,43]. $\;X_{it}$ represents the set of control variables used in all regression models in this paper. α, θ are parameters to be estimated. $\mu_{i}$ and $\tau_{t}$ represent the city-level and time-level fixed effects. $\varepsilon_{it}$ is the standard error term. In this paper, all error terms are clustered at the city level.

Based on the theoretical analysis, we add a quadratic term of fiscal expenditure size in Equation (6) to analyze the possible nonlinear relationship between the expenditure size and GTFP. Considering that the GTFP used in the paper is a continuous cumulative process over time and there may be correlations between its current and earlier periods, the lagging one-stage variable $GTFP_{it-1}$ is added to the Equation (6) [10,15,44]. This leads to the following model:

(7) $$GTFP_{it}=\alpha_{0}+\alpha_{1}GTFP_{it-1}+\alpha_{2}wperexp_{it}+\alpha_{3}wperexp2_{it}+\theta X_{it}+\mu_{i}+\tau_{t}+\varepsilon_{it}\;$$

#### 4.1.2. The Impacts of Fiscal Expenditure Composition on GTFP

Different categories of public expenditures serve different purposes (for example, social expenditures are mainly used to provide basic public services such as education, health care, and pensions, while administrative expenditures are mainly used to maintain the operation of the government). According to the analysis in the previous section, the increase in the proportion of social expenditure and science and technology (S&T) and environmental protection expenditure helps to promote green development, while economic expenditure and administrative expenditure are unfavorable to it. Therefore, we construct a regression model to analyze the relationship between fiscal expenditure composition and GTFP to provide empirical evidence for the results of the theoretical analysis. The specific model is as follows:

(8) $$GTFP_{it}=\alpha_{0}+\alpha_{1}GTFP_{it-1}+\alpha_{2}persco_{it}+\alpha_{3}pereco_{it}+\alpha_{4}perino_{it}+\alpha_{5}pergov_{it}++\theta X_{it}+\mu_{i}+\tau_{t}+\varepsilon_{it}\;$$

where ${\mathrm{persco}}_{\mathrm{it}}$ denotes the proportion of social expenditure to fiscal expenditure and ${\mathrm{pereco}}_{\mathrm{it}}$*,* $perino_{it}$ and $pergov_{it}$ represent the proportion of economic expenditure, S&T and environmental protection expenditure, and administrative expenditure, respectively.

### 4.2. Dynamic Panel Mediation Model

#### Mechanism Analysis of the Impact of Fiscal Expenditure on GTFP

The estimation results of Equations (7) and (8) can help clarify the direction and extent of the impact of changes in the size and composition of fiscal expenditures on GTFP. We further construct dynamic panel mediation models to elucidate the mechanisms of fiscal spending’s impact on GTFP. According to the analysis in Section 2.2, we take human capital accumulation, science and technology innovation, environmental quality, and labor productivity as mediation variables. Human capital accumulation is represented by the number of university students per 10,000 people [45,46,47], and S&T innovation is proxied by the number of green patents granted per 10,000 people [6], environmental quality uses PM2.5 level as a proxy variable [48,49], and labor productivity is expressed as the local GDP divided by the number of local employees [50]. We estimate the following equation using the mediation effects test procedure developed by [51], which mainly referred to the causal steps approach proposed by Baron and Kenny [52]. The specific models are as follows:

(9) $$GTFP_{it}=\alpha_{0}+\alpha_{1}GTFP_{it-1}+\alpha_{2}persco_{it}+\alpha_{3}pereco_{it}+\alpha_{4}perino_{it}+\alpha_{5}pergov_{it}++\theta X_{it}+\mu_{i}+\tau_{t}+\varepsilon_{it}\;\;\;$$

(10) $$mediator_{it}=\alpha_{0}+\alpha_{1}persco_{it}+\alpha_{2}pereco_{it}+\alpha_{3}perino_{it}+\alpha_{4}pergov_{it}+\theta X_{it}+\mu_{i}+\tau_{t}+\varepsilon_{it}\;$$

(11) $$GTFP_{it}=\alpha_{0}+\alpha_{1}GTFP_{it-1}+\alpha_{2}mediator_{it}+\alpha_{3}persco_{it}+\alpha_{4}pereco_{it}+\alpha_{5}perino_{it}+\alpha_{6}pergov_{it}+\theta X_{it}+\mu_{i}+\tau_{t}+\varepsilon_{it}\;\;$$

where Equation (9) is the same as Equation (8), and $mediator_{it}$ represents the four mediation variables mentioned above. 

Equations (7)–(11) all contain lagged terms of the explanatory variables, which represents that these equations are dynamic panel regression models [10]. Since the lagged terms of the explanatory variables may be correlated with the random error terms of the regression models, and there may also be interactions between fiscal expenditure size and structural variables and GTFP, there may be endogeneity among the variables. The estimation results obtained from the traditional static estimation methods may be biased because of the potential endogeneity problems. Therefore, we adopt the generalized method of moments (GMM), which not only alleviates the endogeneity problem of dynamic panel data models but also eliminates the effect of fixed effects and avoids small sample errors (for one thing, system GMM can utilize more information than difference GMM. It can also estimate variables that do not vary with time, and the accuracy of the estimation results is higher. For another thing, two-step GMM is more robust and efficient than a one-stage estimate. However, the two-step estimate is prone to a severe underestimation of the standard errors of the estimated coefficients when the sample is small. In this paper, the standard error estimates are corrected using the method proposed by Windmeijer [53]). To be more specific, this paper uses a two-stage system GMM. Considering that the key to obtaining consistently estimated coefficients for two-step system GMM is the selection of valid instrumental variables and the absence of second-order autocorrelation in the residual terms, we use the Hansen test to determine the validity of the selected instrumental variables and the AR test to determine whether there is autocorrelation in the random error term after the first-order difference. In addition, to ensure the consistency of the estimation method, we also use a two-step system GMM model for the estimation of Equation (10).

### 4.3. Dynamic Threshold Panel Model 

#### The Impact of Fiscal Expenditure Composition on the Relationship between Fiscal Expenditure and GTFP

Changes in the proportion of a particular type of fiscal expenditure may affect the relationship between the fiscal expenditure size and GTFP (for example, if the Chinese government expands fiscal spending while maintaining a high proportion of social spending, the resulting impact on GTFP may be different from the impact of fiscal spending expansion with a relatively low share of social spending). Therefore, we develop a dynamic threshold panel model to analyze the effect of changes in the proportion of each category of public expenditure on the green growth effect of the fiscal expenditure size’s expansion. The specific model is as follows:

(12) $$GTFP_{it}=\alpha_{0}+\alpha_{1}GTFP_{it-1}+\alpha_{2}wperexp_{it}+\alpha_{3}wperexp2_{it}\cdot Q\left(Z_{j}\leq \gamma_{j}\right)+\alpha_{4}wperexp2_{i,t}\cdot Q\left(Z_{j}>\gamma_{j}\right)+\theta X_{it}+\mu_{i}+\tau_{t}+\varepsilon_{it}\;$$

where Q(·) is an indicator function, $Z_{j}\left(j=1,2,3,4\right)$ denotes the regime-switching variables, including the fiscal expenditure composition variables, i.e., $persco_{it},\;pereco_{it},\;perino_{it}$, and $\;pergov_{it}$. $\gamma_{j}\left(j=1,2,3,4\right)$ represents the threshold parameter. The estimation results of dynamic threshold panel models obtained from static methods are biased. Therefore, we refer to the method proposed by Dang et al. [54] and improved by Wu et al. [55] (this method uses a grid search algorithm over the range between the 15th and 85th percentiles of the regime-switching variable to calculate the threshold value. Then, the Wald statistic, whose gradual distribution can be obtained through the bootstrap method, is used to determine whether the threshold effect is significant or not. The smaller the corresponding probability is, the more significant the threshold effect. Finally, the two-step system GMM is used to estimate the coefficients of each variable) to estimate Equation (12), which can endogenously determine the threshold values based on the characteristics of the regime-switching variables and solve the potential endogeneity problem.

### 4.4. Data Source

We include the same set of explanatory variables in all regression models to reduce model specification errors and to capture city-level characteristics. Drawing on existing studies [6,10,20], we choose the level of infrastructure (road), the level of financial development (mon), the degree of opening up (fdi), the level of economic development (pgdp), the industrial structure (sec), and the degree of fiscal decentralization (gov) as explanatory variables. The sample period of this paper ranges from 2010 to 2018, and, due to the lack of data in some cities, the data of each period contain 275 China’s prefecture-level cities. We present the definitions, measures, and descriptive statistics of the dependent variables, core independent variables, and control variables in Table 5. Data for these variables are from the China Urban Statistical Yearbook, China Urban Construction Statistical Yearbook, the EPS database, economic and social development reports of each city, and the statistical yearbooks published on the official websites of each city’s statistical bureaus.

## 5. Empirical Results

### 5.1. The Results of the Dynamic Models Estimation

#### 5.1.1. The Impacts of Fiscal Expenditure Size on GTFP

We present the results of Equation (7) using the two-step system of GMM in column (4) of Table 6. The Hansen test as well as AR(2) test cannot be rejected at the 10% significance level, indicating that the instrumental variables we selected are valid and that there is no autocorrelation in the first difference of the random error terms [56,57].

The results in Table 6 indicate that the estimated coefficient for the fiscal expenditure size is positive, and the coefficient for its squared term is negative, both of which are significant at the 1% level. This suggests an inverted U-shaped relationship between fiscal expenditure and GTFP, which is consistent with the analysis in the second section and with the findings of Armey [58] and Chen and Lee [59]. In other words, all else being equal, there may be an optimal size of fiscal expenditure that results in the highest level of GTFP in China. Until this size is reached, expanding fiscal expenditure size will be beneficial to improving GTFP. However, this positive effect decreases at the margin as the expenditure size increases, eventually declining to zero when the optimal expenditure size is reached. We draw on Wu et al. [60] to use the OLS model, FE model, and two-step system GMM to repeat the estimation of Equation (6) to ensure the robustness of the inverted U-shaped relationship. The estimation results in columns (1) to (3) indicate that the significance and sign of the coefficients of the fiscal expenditure size and its squared term are almost the same using different estimation methods, demonstrating the robustness of the inverted U-shaped relationship.

We use estimated coefficients in column (4) to calculate the turning point of the inverted U-shaped relationship between fiscal expenditure size and GTFP, which is 75,000 yuan per capita. The only two cities, Lanzhou and Nantong, exceeded this point in 2018. This phenomenon suggests that the vast majority of prefecture-level cities in China can improve GTFP and promote green economic development by expanding the fiscal expenditure size. It also suggests that the Chinese government’s expansionary fiscal spending preferences are somewhat justified. Moreover, we estimated Equation (7) without the squared term. The results in column (5) show that the coefficient of the fiscal expenditure size is significantly positive at the 1% level, which empirically proves the robustness of this finding. 

To ensure that the nonlinear relationship between expenditure size and GTFP is robust to different specifications of public spending size, we further constructed another variable, which is $expgdp_{it}$, measured as the size of fiscal spending divided by local GDP, and re-estimated Equation (7). Column (6) indicates that the sign and significance of the coefficients of alternative measurements of expenditure size and its quadratic term remain unchanged, proving that the inverted U-shaped relationship is robust. In addition, the coefficients of the first-order lagged terms of GTFP in columns (3) to (6) are all significantly positive, indicating that the increase in GTFP in the previous period is conducive to the improvement in the economic growth quality in the current period. This suggests that China’s green and high-quality development has strong inertia, consistent with conclusions obtained from the analysis in Section 3.2 above and the findings in Wu et al. [15].

#### 5.1.2. The Impacts of Fiscal Expenditure Composition on GTFP 

We drew on the approach used in Jia et al. [14] when estimating Equation (8). Specifically, we first estimated this equation using the two-step system of GMM. Afterward, we validated the robustness of the regression results by placing each expenditure composition variable in four regression equations for estimation. Table 7 shows that the estimated coefficients of the expenditure composition variables and other explanatory variables in columns (2) to (5) are very close to those in column (1), which indicates that the estimation results in this section are robust. We also conducted sensitive analysis by constructing alternative measurements of public expenditure composition, namely, using each category of public expenditure scaled by local GDP as independent variables. The estimation results shown in column (6) are consistent with those in column (1). In addition, all six regression models pass the second-order autocorrelation test. The results of the Hansen test also show that the original hypothesis of the validity of the instrumental variables cannot be rejected. Therefore, the whole model is reasonably set up, and the instrumental variables chosen are valid.

We regard column (1) as the benchmark result, which shows that an increase in the proportion of social expenditure and the proportion of expenditure on science and technology (S&T) and environmental protection can improve GTFP. On the contrary, an increase in the share of economic expenditure or administrative expenditure is not beneficial to GTFP, provided that the public expenditure size remains unchanged. These results are consistent with the previous analysis.

At present, China has entered the stage of high-quality economic development [10]. The limitations of the previous high-consumption, high-pollution economic development strategy gradually became apparent. Therefore, an increase in the proportion of economic expenditure will not be conducive to GTFP. In order to achieve sustained high-quality growth, the Chinese government needs to promote a shift from a factor-driven to an innovation-driven economic growth and focus on improving the quality of people’s lives and ecological environment. Social expenditure and expenditure on S&T and environmental protection are more in line with the connotation of high-quality economic development than economic expenditure, and increasing the proportion of these two types of expenditure is conducive to the rise of GTFP. In addition, administrative expenditure does not directly contribute to economic development and ecological quality improvement. Excessive administrative expenditure means that government operations are inefficient and costly, which may obstruct green and high-quality economic developmen [14]. Therefore, increasing the proportion of administrative expenditure will not be conducive to improving GTFP.

### 5.2. The Results of the Dynamic Panel Mediation Models’ Estimation

#### Mechanism Analysis of the Impact of Fiscal Expenditure on GTFP

The results in Table 7 reveal that the impacts of changes in the proportion of different types of fiscal expenditures on GTFP are different. However, the results do not reflect the mechanism of these heterogeneous impacts. Therefore, we further construct dynamic panel mediation models to clarify the paths through which the composition of fiscal expenditure affects GTFP. We estimated Equations (9)–(11) using the two-step system GMM and present the results in Table 8. The results in all columns pass the Hansen test and AR(2) test, which proves that the models we set are reasonable and valid. The existence of the mediation effects requires the following four conditions to be satisfied [51]:

1. Before including the mediation variables in the regression model, the effect of the core independent variables on the dependent variables is statistically significant.
2. The effects of independent variables on the mediation variables are statistically significant.
3. After including the mediation variables, the effects of these variables on the dependent variables are statistically significant.
4. After including the mediation variables, the effects of the core independent variables on the independent variables weaken.

The estimated results of the four sets of mediation models we obtained fully satisfy the above four conditions. Next, we interpret the results one by one.

Columns (1)–(3) in part a of Table 8 present the estimated results of the regression model with human capital accumulation as the mediation variable. Column (1) shows that an increase in the level of human capital can significantly improve GTFP. Human capital accumulation is fundamental to technological progress. Moreover, an increase in human capital helps improve society’s overall resource allocation and management capacity. Therefore, improving the human capital level can enhance GTFP [10].

In column (2) of part a of Table 8, we can see that the increase in the proportion of social expenditure can significantly raise the number of college students per 100 people, which suggests that the government’s educational spending can effectively motivate individuals to receive high-level education, thus promoting human capital accumulation [10]. Compared with the social expenditure, an increase in the proportion of S&T and environmental expenditure has a higher positive effect. This may be due to the fact that S&T expenditure can significantly increase the overall level of scientific and technological knowledge stock in society, which can more directly stimulate the increase in human capital level. Our results also indicated that the expansion of administrative expenditure could also increase the level of human capital, which seems to be inconsistent with our theoretical analysis. We argue that this is reasonable as the government can remove institutional barriers to human resource allocation by increasing general public service expenditure [61], which in turn can optimize the human capital allocation structure. The increase in the share of economic expenditure has a significant inhibitory effect on human capital. The primary function of economic expenditures is to compensate for market failures and maintain market resource allocation, and expanding the share of economic expenditures has a crowd-out effect on other public spendings [18].

Columns (4)–(6) present the estimation results of the regression model with science and technology innovation (STI) as the mediating variable. According to column (4), STI has a significant positive effect on GTFP. For one thing, firms improve the input–output ratio and reduce the pollution emissions of economic activities mainly by developing and applying new technologies. For another thing, STI implies technological progress, which is one of the components of GTFP [35]. Therefore, the increase in the STI level can improve GTFP.

The results in column (5) suggest that an increase in the share of social expenditures can promote STI. This is because social spending has a significant human capital accumulation effect, and human capital is considered to be an important way to incur STI. The positive impact of expanding the proportion of S&T and environmental expenditures on GTFP is more significant than that of social expenditures. This may indicate that government spending on S&T can directly support economy-wide R&D spending and innovation compared to social spending [19]. Expenditures on energy conservation and environmental protection can directly compensate for green innovation, energy-saving technologies, and ecological protection, which have strong green development attributes [23,25]. Economic and administrative expenditures do not directly support innovation in science and technology. Increasing the proportion of both will crowd out social and environmental expenditures, so increasing the proportion of both will inhibit GTFP’s rise.

Columns (7) to (9) present the estimation results of the model with environmental quality as the mediation variable. The results in column (7) indicate that the deterioration of environmental quality significantly impairs GTFP. Green development requires a balance between economic growth, resource conservation, and environmental friendliness, and the development strategy at the cost of environmental quality is contrary to the connotation of green development [62]. Therefore, the damage to the environment and ecology will directly reduce GTFP.

Column (8) shows that increasing social expenditures is not conducive to environmental quality improvement. Social expenditure can influence STI by promoting human capital accumulation. Technologies related to cleaner production and energy conservation are mainly applied by firms to improve environmental quality. However, this indirect impact on environmental quality may have a certain lag [63], resulting in an insignificant green effect of social spending. In contrast, S&T expenditure can directly stimulate green innovation [26]. Energy conservation and environmental protection spending can also compensate for ecological and environmental management behaviors more straightforwardly than social expenditure. Therefore, expanding the proportion of S&T and environmental protection expenditure can significantly improve environmental quality.

An increase in economic spending at the 1% level is beneficial to environmental quality, which seems like a counterintuitive conclusion. One reason for this may be that the Chinese government has been optimizing the composition and direction of its fiscal expenditure on agriculture, forestry, and water affairs in recent years to promote a low-carbon transition in agriculture [14]. Chinese agriculture has been characterized by high consumption, high emissions, and high pollution for quite a long time [64]. In recent years, the Chinese government has actively promoted economical and environmentally friendly agricultural technologies and increased the efficiency of energy use and the proportion of clean energy in agriculture. With unremitting efforts, the growth rate of China’s agricultural carbon emissions has gradually slowed down and tended to reach its peak [65]. Therefore, the expansion of economic expenditures can improve the environmental quality to some extent. Finally, the increase in the share of administrative expenditure is not conducive to environmental quality improvement due to the strong crowding-out effect of administrative expenditure on scientific and environmental expenditures and economic expenditures.

Columns (10)–(12) present the estimation results with labor productivity as a mediation variable. According to column (10), an increase in labor productivity can significantly improve GTFP. Increasing labor productivity implies that more output can be obtained per unit of time for the same labor, i.e., reaching a higher input–output ratio. Moreover, from the perspective of the calculation process of GTFP, a higher input–output ratio symbolizes a higher GTFP.

We can see from column (11) in part b of Table 8 that social expenditure can significantly improve labor productivity. Social spending can promote the diffusion of advanced production technologies, improve practitioners’ knowledge and operational proficiency, provide high-quality labor for economic activities, and increase labor productivity [9]. The positive effect of increasing the share of expenditure on S&T and environmental protection on labor productivity is greater than that of social expenditure, which indicates that the innovation of production technology is the fundamental driving force to production efficiency improvement. In addition, moderately reducing economic and administrative expenditures may be another essential way to improve labor productivity.

We clarify in this section the paths through which fiscal expenditure affects GTFP. Specifically, social spending positively affects GTFP through promoting human capital accumulation, scientific and technological innovation, and increasing labor productivity. Economic expenditure mainly improves GTFP by improving environmental quality. Scientific and environmental expenditure has positive effects on GTFP through all four channels. Administrative expenditure can improve GTFP by increasing the level of human capital. These results can be regarded as an essential complement to the current literature where the influencing channels of public expenditure composition have been explored less.

### 5.3. The Results of the Dynamic Threshold Panel Models’ Estimation

#### The Impact of Fiscal Expenditure Composition on the Relationship between Fiscal Expenditure and GTFP

We have explored in detail the impacts of fiscal expenditure on GTFP and revealed the possible mechanisms of these impacts. In analyzing how changes in the composition of fiscal expenditure affect GTFP, we assumed a constant size of expenditure. Therefore, the results obtained in the previous section can be referred to as static effects of public expenditure composition. In this section, we construct a dynamic threshold panel model with four categories of fiscal expenditure as regime-switching variables to analyze the dynamic effects of public spending composition, i.e., how changes in spending composition affect the “inverted U-shaped” relationship between expenditure size and GTFP. Similar evaluation is still lacking among the existing studies. Specifically, we estimate Equation (12) by drawing on the methodology used in Dang et al. [54] and Wu et al. [55] and present the results in Table 9 and Table 10, respectively.

Table 9 shows the threshold values and confidence intervals for the four types of public expenditure composition variables. The *p*-values of Wald statistics indicate that all the threshold effects are significant at the 1% significance level. Therefore, we can conclude that changes in the share of different fiscal expenditures can affect the inverted U-shaped relationship between the size of fiscal expenditures and GTFP.

The results of the Hansen test, as well as the AR(2) test in Table 10 columns (1)–(4), are not rejected at the 10% significance level, indicating that the instrumental variables we selected are valid and the first difference of the random error terms is not second-order autocorrelated. The results in column (1) show that when the share of social expenditures reaches the threshold (c = 52.5%), the regression coefficient of the squared term of fiscal expenditure size changes, indicating variation in the inverted U-shaped relationship between fiscal expenditure size and GTFP. Specifically, when the share of social expenditure is less than the threshold, the positive effect of fiscal expenditure expansion on GTFP disappears after per capita fiscal expenditure reaching 81,000 yuan. However, when the percentage of social spending exceeds the threshold, the turning point increases from 81,000 yuan to 109,000 yuan, with a percentage of 34.6%, implying a more extensive scope for fiscal spending to improve GTFP. Based on the empirical results in the previous sections, it is clear that social spending can promote human capital accumulation, technological R&D innovation, and labor productivity. Therefore, it is more beneficial for the government to expand its fiscal expenditure while maintaining a high proportion of social expenditure for China’s long-term green development.

Column (2) results suggest that the government should pay attention to controlling the proportion of economic expenditure when expanding the size of expenditure. Appropriate economic spending can compensate for market failures, maintain the allocation of market resources and adjust the operation of the national economy, which has a significant boosting effect on GTFP. However, as economic spending is inclined to factor inputs to drive economic development, the expansion of fiscal spending with economic spending exceeding the threshold value (c = 13.2%) is not conducive to China’s long-term development goal of green transformation [18].

The results in column (3) show that the expansion of public expenditure after the share of S&T and environmental protection expenditure reaches the threshold (c = 3.5%) has a more lasting boost to GTFP than when the threshold is not reached. S&T spending can facilitate the shift in China’s economic growth model from factor-driven to innovation-driven. Moreover, spending on energy efficiency and environmental protection can compensate for ecological and environmental management and promote environmentally friendly production technologies [21,25]. Therefore, the government should ensure a higher share of S&T and environmental expenditure when expanding the size of fiscal expenditure, which is more beneficial to China’s long-term green development. Moreover, when the share of spending on S&T and environmental protection is below the threshold, the turning point of the “inverted U-shaped” relationship between fiscal expenditure and green total factor productivity is relatively low. This phenomenon confirms that the old path of relying on factor inputs to drive economic growth is not sustainable.

The results in column (4) are interesting, which show that the positive effect of fiscal expenditure on GTFP is limited when the share of administrative expenditure is below the threshold (c = 13.4%). However, when it is above the threshold, the contribution of fiscal expenditure to green development is extended, which may be contradictory to current studies, such as those of Lopez et al. [7] and Jia et al. [14]. One possible explanation for this is that the increase in expenditure on defense and public security provides a strong material basis for securing national territory and sovereignty as well as social stability. Moreover, it is only when the country is stable and socially stable that people’s livelihoods improve and the country’s economic development is guaranteed. In addition, an appropriate size of public service expenditure can improve the wages and benefits of public servants, increase their motivation and improve the efficiency of government operations, and optimize the allocation of human capital to a certain extent [9]. This result, in fact, suggests something to the Chinese government, namely, not to over-compress administrative expenditure and that an appropriate proportion of administrative expenditure is more conducive to China’s long-term green development.

## 6. Conclusions and Policy Implications

In this paper, we elaborate on the theoretical mechanism by which public expenditure size and composition affect green development. Afterward, urban green total factor productivity is measured using data from 275 prefecture-level cities in China from 2010 to 2018. We treat it as a proxy variable for green development in China and perform the two-stage GMM system on dynamic panel models to examine the impact of the size and composition of public spending on green growth. In order to disclose the possible mechanisms through which public expenditure affects GTFP, dynamic panel mediation models are further constructed and estimated. Finally, we used dynamic threshold panel models to analyze the impacts of changes in public expenditure composition on the inverted U-shaped relationship between fiscal expenditure size and GTFP.

The main findings of this paper are as follows: (1) China achieved green development between 2010 and 2018, with GTFP showing an oscillating growth trend of rising, then falling, then rising again. The four main economic regions in China have also achieved green development. The eastern region mainly relied on technological progress to promote green growth, while the northeast, central, and western regions were driven by pure technical efficiency and scale efficiency. (2) Currently, the expansion of fiscal spending by the Chinese government continues to promote green growth. At the same time, there is a clear, inverted U-shaped relationship between fiscal expenditure and GTFP. (3) There are significant differences in the impacts of different types of fiscal expenditures on GTFP, with social and S&T and environmental protection expenditures having a particularly significant boost to GTFP. In contrast, economic and administrative expenditures inhibit the improvement of GTFP. (4) Human capital accumulation, technological innovation, environmental quality, and labor productivity are essential mediators of fiscal expenditure’s green effect. (5) The turning point of the inverted U-shaped relationship between fiscal expenditure size and GTFP can be influenced by fiscal expenditure composition. When social spending reaches its threshold value, i.e., when the share of social spending exceeds 52.5% of total public spending, the turning point of the inverted U-shaped curve expands from 81,000 yuan to 109,000 yuan per capita, which is an increase of 34.6%, indicating that fiscal spending has more room for expansion to promote green development. S&T and environmental protection expenditures and administrative expenditures have similar effects when they exceed their respective thresholds, i.e., 3.5% and 13.4%. However, when the proportion of economic expenditure surpasses 13.2%, the turning point narrows significantly, from 73,000 yuan to 38,000 yuan per capita, which is a decrease of 48.0%, also indicating that a large proportion of economic expenditures is not conducive to expanding the green effect of fiscal expenditure.

Based on these findings, the following relevant and straightforward policy implications can be derived: (1) there is an optimal size of public expenditure with tax financing as the main source. For developing countries with a high overall tax burden, the contribution of fiscal expenditure to GTFP may be close to a critical value. In other words, relying solely on the expansion of fiscal expenditure size to promote green and high-quality transformational development is feasible but not sustainable, as it may impose a heavy burden on economic growth and environmental governance. (2) Optimizing fiscal expenditure composition may be the preferred strategy for promoting green development compared to expanding the size of fiscal expenditure. Specifically, for developing countries, which are mostly developmental governments, increasing the proportion of social spending and spending on environmental protection and science and technology may better accelerate their transformation towards green and innovation-driven economies. Moreover, improving the expenditure structure can also increase the marginal benefits of the expansion of public expenditure size. Meanwhile, the transformation of government governance concepts and functions, the correct handling of the relationship between government and the market, and the gradual withdrawal of government from competitive areas, as well as increased supervision and restraint of government, are essential guarantees of the effectiveness of expenditure structure optimization. (3) Economic and administrative expenditures generally cannot promote green development, but governments can amplify their effects on human capital accumulation, environmental quality improvement, technological innovation, and labor productivity enhancement. These are the four main channels of fiscal expenditure promoting green economic development.

This paper extends the existing research, helping to understand the relationship between public spending and green development from a more comprehensive perspective. At the same time, there are limitations and room for further extension of this paper. For example, the DEA-SBM model and GML index used in this paper to measure GTFP are typical non-parametric methods. The parametric methods, such as the Stochastic Frontier Model, can also be used for the calculation of the level of green growth. Moreover, we regard electricity consumption as city-level energy inputs, while using specific energy data such as oil, natural gas, and petroleum consumption in GTFP’s evaluation may derive more accurate results. There is also room for improvement of the public expenditure measurement, as the implicit fiscal expenditure and quasi-fiscal expenditure commonly exist in developing countries, including China, which may need further expansion in subsequent studies. Moreover, how to consider the spatial relationship between fiscal expenditure and green development remains a complex problem, and the findings may be more affluent after analyzing it.

## Figures and Tables

**Table 1:** Size and proportion of various types of public expenditure in Chinese local governments.

Year	Social Expenditures		Economic Expenditures		S&T and Environmental Protection Expenditures		Administrative Expenditures	
	Size	Proportion	Size	Proportion	Size	Proportion	Size	Proportion
	100 Million yuan	%	100 Million yuan	%	100 Million yuan	%	100 Million yuan	%
2010	34,600.26	46.83	11,740.58	15.89	3961.38	5.36	13,142.24	17.79
2011	45,268.83	48.82	16,687.68	18.00	4452.67	4.80	15,352.03	16.55
2012	54,515.73	50.86	18,803.96	17.54	5142.01	4.80	17,630.27	16.45
2013	60,510.30	50.53	21,448.47	17.91	6050.20	5.05	19,243.42	16.07
2014	67,192.69	52.00	23,303.42	18.03	6348.69	4.91	19,096.54	14.78
2015	79,154.02	52.65	28,144.98	18.72	7786.66	5.18	20,288.28	13.50
2016	88,022.30	54.89	27,494.88	17.15	8317.19	5.19	22,871.44	14.26
2017	96,372.77	55.63	27,897.81	16.10	9706.79	5.60	25,851.23	14.92
2018	103,273.08	54.88	30,462.34	16.19	11,076.43	5.89	28,610.98	15.20

Data source: China Statistical Yearbook (2011–2019).

**Table 2:** The definitions and descriptive statistics of the input and output indicators for GTFP’s calculation.

Indicators		Definition	Unit	Mean	SD	Min	Max
	Workforce (L)	Persons employed in urban units at year-end	Person	51.15	56.80	5.01	613.50
Inputs	Capital Stock (K)	calculated through the perpetual inventory method	Billion Yuan	491.13	481.03	26.44	3382.64
	Water Supply (W)	-	Million tons	140.46	240.56	2.020	2288.50
	Electricity Supply (E)	-	Million kwh	10,771.95	14,081.44	97.54	156,248.97
Desirable outputs	GDP	Gross Regional Product (base period 2000)	Billion Yuan	1238	1399	68.78	12,870
	Green Coverage (G)	Green-covered area as % of built-up area	Percentage	38.55	8.06	0.36	95.25
Undesirable outputs	Industrial Waste (IW)	Volume of industrial waste water discharged	Ten thousand tons	6323	7589	7	93,814
	Sulfur Dioxide Emissions (SDE)	Volume of Industrial sulfur dioxide emission	Ton	42,978	43,836	0	496,377
	Soot Emission (SE)	Volume of industrial soot (dust) emission	Ton	35,191	151,948	34	5,168,812

**Table 3:** National average values of GTFP and its composition.

Year	National Average			
	GTFP	GTECH	PGEFFCH	SGEFFCH
2010	1.070	1.009	1.062	1.020
2011	1.025	0.944	1.104	1.019
2012	1.024	0.961	1.085	1.017
2013	1.007	0.872	1.166	1.024
2014	0.940	0.884	1.088	1.008
2015	0.928	0.839	1.153	0.998
2016	1.036	0.946	1.090	1.031
2017	1.083	0.860	1.232	1.051
2018	1.147	0.931	1.213	1.057

**Table 4:** Regional average values of GTFP and its composition.

Year	Northeast China Average				East China Average			
	GTFP	GTECH	PGEFFCH	SGEFFCH	GTFP	GTECH	PGEFFCH	SGEFFCH
2010	1.079	0.971	1.093	1.031	1.078	1.098	1.026	0.979
2011	1.069	0.909	1.174	1.047	0.994	1.035	1.005	0.971
2012	1.056	0.937	1.127	1.046	0.986	1.038	1.017	0.950
2013	1.158	0.863	1.285	1.086	0.955	0.943	1.094	0.955
2014	0.935	0.858	1.109	1.029	0.898	0.932	1.027	0.963
2015	0.911	0.811	1.163	1.012	0.904	0.889	1.103	0.951
2016	0.998	0.907	1.107	1.039	1.016	1.009	1.030	0.999
2017	1.129	0.820	1.315	1.071	1.080	0.936	1.155	1.015
2018	1.150	0.849	1.305	1.065	1.128	1.067	1.069	1.036
**Year**	**Central China Average**				**West China Average**			
	**GTFP**	**GTECH**	**PGEFFCH**	**SGEFFCH**	**GTFP**	**GTECH**	**PGEFFCH**	**SGEFFCH**
2010	1.011	0.969	1.059	1.008	1.114	0.999	1.071	1.063
2011	0.978	0.919	1.124	0.985	1.061	0.915	1.114	1.074
2012	0.988	0.936	1.101	1.002	1.066	0.934	1.096	1.070
2013	0.924	0.840	1.130	1.006	0.989	0.842	1.158	1.049
2014	0.920	0.882	1.049	1.009	1.007	0.864	1.167	1.029
2015	0.891	0.828	1.144	0.980	1.006	0.827	1.202	1.047
2016	1.031	0.937	1.082	1.027	1.098	0.931	1.141	1.061
2017	1.070	0.841	1.265	1.024	1.053	0.841	1.195	1.092
2018	1.184	0.900	1.279	1.048	1.127	0.908	1.200	1.079

**Table 5:** Variables used in the empirical analysis and their definitions.

Variable	Mean	SD	Min	Max	N
Green total factor productivity, gtfp	1.021	0.262	0.362	2.311	2475
Fiscal expenditure per capita, wperexp, 10 thousand yuan	0.648	0.944	0.023	13.303	2456
The square of wperexp, wperexp2	1.311	7.488	0.001	176.958	2456
The number of university students per 10,000 people, pouni	1.836	2.431	0.006	13.112	2426
The number of green patents granted per 10,000 people, pogreenino	0.477	1.058	0.000	18.396	2473
PM2.5, pm25, μg/m³	42.814	19.514	4.134	110.121	2475
The local GDP divided by the number of local employees, labor	24.583	10.322	0.633	140	2473
The proportion of social expenditure in total fiscal expenditure, persco	0.501	0.072	0.045	0.958	2444
The proportion of economic expenditure in total fiscal expenditure, pereco	0.162	0.052	0.013	0.691	2425
The proportion of environmental protection and S&T expenditure in total fiscal expenditure, perino	0.045	0.023	0.002	0.263	2437
The proportion of administrative expenditure, pergov	0.157	0.037	0.034	0.402	2375
Area of city paved roads per capita at year-end, road, 10,000 sq.m	6.351	11.710	0.102	162.383	2416
The proportion of loans of national banking system at year-end in GDP, mon	0.907	0.549	0.118	7.450	2473
The proportion of foreign capital actually utilized in GDP, fdi	0.018	0.0177	0.000	0.210	2348
GDP per capita, pgdp, 10,000 yuan	2.812	1.796	0.352	17.059	2468
The proportion of employees in the secondary industry to all employees, sec	0.453	0.142	0.045	0.844	2473

**Table 6:** Estimation results of public expenditure scale on GTFP.

	OLS	Fixed Effect	Two-Step SYS-GMM	Two-Step SYS-GMM	Two-Step SYS-GMM	Two-Step SYS-GMM
	(1)	(2)	(3)	(4)	(5)	(6)
VARIABLES	GTFP	GTFP	GTFP	GTFP	GTFP	GTFP
L. **GTFP**				0.339 ***	0.303 ***	0.286 ***
				(0.004)	(0.004)	(0.002)
wperexp	0.023 *	0.099 **	0.149 ***	0.103 ***	0.056 ***	
	(0.094)	(0.015)	(0.000)	(0.010)	(0.008)	
wperexp2	−0.004 ***	−0.008 ***	−0.009 ***	−0.007 ***		
	(0.005)	(0.002)	(0.000)	(0.009)		
expgdp						2.414 ***
						(0.008)
Expgdp2						−4.664 ***
						(0.005)
road	0.001	−0.000	−0.005 **	−0.003 *	−0.002 *	0.001
	(0.135)	(0.972)	(0.026)	(0.060)	(0.062)	(0.326)
mon	−0.033 ***	−0.026	−0.029	−0.033 **	−0.030 **	−0.018
	(0.001)	(0.208)	(0.125)	(0.038)	(0.049)	(0.181)
fdi	−0.209	1.124 **	−0.095	0.048	0.096	−0.325
	(0.482)	(0.011)	(0.916)	(0.901)	(0.808)	(0.624)
pgdp	0.041 ***	0.060 ***	0.041 ***	0.037 ***	0.040 ***	0.040 ***
	(0.000)	(0.001)	(0.001)	(0.000)	(0.000)	(0.000)
sec	−0.286 ***	−0.453 ***	−0.230 **	−0.461 **	−0.404 **	−0.001
	(0.000)	(0.000)	(0.011)	(0.011)	(0.029)	(0.239)
gov	0.001	0.020	−0.027	0.025**	−0.001	−0.002
	(0.863)	(0.110)	(0.192)	(0.013)	(0.940)	(0.825)
Constant	1.086 ***	1.048 ***	1.088 ***	0.735 ***	0.766 ***	0.506 ***
	(0.000)	(0.000)	(0.000)	(0.000)	(0.000)	(0.003)
Observations	2.268	2.268	1.978	2.008	2.008	1.978
Adjusted R²	0.132	0.218				
City FE	NO	YES	YES	YES	YES	YES
Time FE	YES	YES	YES	YES	YES	YES
AR(2)			−1.231	1.059	1.003	0.848
			[0.218]	[0.290]	[0.316]	[0.396]
Hansen test			8.868	14.654	9.259	4.468
			[0.354]	[0.686]	[0.753]	[0.614]
Number of cities	270	270	269	270	270	269

Note: Robust and cluster standard errors in parentheses and *p*-value in brackets. *** *p* < 0.01, ** *p* < 0.05, * *p* < 0.1.

**Table 7:** Estimation results of the impact of public expenditure composition on GTFP.

	(1)	(2)	(3)	(4)	(5)	(6)
VARIABLES	GTFP	GTFP	GTFP	GTFP	GTFP	GTFP
L. **GTFP**	0.630 ***	0.238 **	0.277 **	0.647 ***	0.620 ***	0.610 ***
	(0.000)	(0.045)	(0.017)	(0.000)	(0.000)	(0.000)
persco	0.852 **	1.129 ***				
	(0.023)	(0.009)				
pereco	−0.408 **		−0.914 **			
	(0.027)		(0.048)			
perino	1.397 ***			0.917 **		
	(0.005)			(0.010)		
pergov	−1.100 **				−0.776 **	
	(0.014)				(0.048)	
scogdp						0.483 *
						(0.078)
ecogdp						−1.090 *
						(0.079)
inogdp						1.548 **
						(0.025)
govgdp						−1.109 *
						(0.089)
road	−0.000	0.001	0.000	−0.001	−0.002	0.001
	(0.860)	(0.215)	(0.650)	(0.525)	(0.349)	(0.495)
mon	−0.084 ***	−0.063 ***	−0.073 **	−0.034 *	−0.087 ***	−0.088 ***
	(0.008)	(0.009)	(0.022)	(0.066)	(0.005)	(0.005)
fdi	1.286*	1.250	1.058	−0.178	−0.190	0.774
	(0.087)	(0.193)	(0.240)	(0.554)	(0.570)	(0.202)
pgdp	0.005	0.034 ***	0.032 ***	0.040 ***	0.065 **	0.022 ***
	(0.684)	(0.000)	(0.001)	(0.007)	(0.036)	(0.008)
sec	−0.215 ***	−0.276 ***	−0.355 ***	−0.272 ***	−0.318 **	−0.003 ***
	(0.001)	(0.001)	(0.000)	(0.001)	(0.015)	(0.000)
gov	−0.031	0.006	−0.133 *	0.004	−0.003	−0.053
	(0.690)	(0.878)	(0.075)	(0.744)	(0.534)	(0.448)
Constant	0.336	0.302	1.154 ***	0.413 ***	0.601 ***	0.638 ***
	(0.195)	(0.220)	(0.000)	(0.000)	(0.000)	(0.000)
Observations	1.927	1.944	1.956	1.991	1.958	1.929
City FE	YES	YES	YES	YES	YES	YES
Time FE	YES	YES	YES	YES	YES	YES
AR(2)	1.463	0.402	1.142	1.617	1.579	1.630
	[0.144]	[0.687]	[0.254]	[0.106]	[0.114]	[0.103]
Hansen test	34.427	17.354	19.632	10.942	7.239	38.170
	[0.154]	[0.363]	[0.354]	[0.205]	[0.299]	[0.209]
Number of cities	269	268	269	270	269	269

Note: Robust and cluster standard errors in parentheses and p-value in brackets. *** *p* < 0.01, ** *p* < 0.05, * *p* < 0.1.

**Table 8:** (**a**) Estimation results of the dynamic panel mediation models. (**b**) Estimation results of the dynamic panel mediation models.

(**a**)						
	**(1)**	**(2)**	**(3)**	**(4)**	**(5)**	**(6)**
**MEDIATORS**	**Human Capital Accumulation**			**Technological Innovation**		
**VARIABLES**	**GTFP**	**Pouni**	**GTFP**	**GTFP**	**Pogreenino**	**GTFP**
L. **GTFP**	0.660 ***		0.305 ***	0.655 ***		0.307 ***
	(0.000)		(0.003)	(0.000)		(0.000)
pouni	0.018 *		0.024 ***			
	(0.085)		(0.002)			
pogreenino				0.037 *		0.048 **
				(0.096)		(0.013)
persco	0.842 *	8.787 ***		0.768 **	2.405 ***	
	(0.093)	(0.000)		(0.020)	(0.001)	
pereco	−0.279 *	−12.712 ***		−0.323 **	−2.798 **	
	(0.072)	(0.001)		(0.039)	(0.034)	
perino	0.882 ***	10.259 *		1.384 ***	5.927 ***	
	(0.007)	(0.059)		(0.002)	(0.007)	
pergov	−1.384 ***	11.610 **		−1.025 **	−2.088 *	
	(0.002)	(0.016)		(0.031)	(0.093)	
road	−0.001	0.029	−0.002	−0.001	0.055 **	0.001
	(0.509)	(0.115)	(0.162)	(0.144)	(0.017)	(0.279)
mon	−0.065 ***	1.862 ***	−0.085 ***	−0.071 **	−0.213	−0.060 **
	(0.005)	(0.000)	(0.003)	(0.013)	(0.153)	(0.014)
fdi	0.015	9.001 **	−0.223	2.707 ***	−8.464 **	0.053
	(0.966)	(0.013)	(0.523)	(0.007)	(0.039)	(0.873)
pgdp	0.001	0.695 ***	0.028 ***	−0.009	0.513 ***	0.015
	(0.966)	(0.001)	(0.001)	(0.587)	(0.000)	(0.110)
sec	−0.053	−5.236 ***	−0.187 ***	−0.193 ***	−0.941	−0.250 ***
	(0.485)	(0.000)	(0.009)	(0.005)	(0.147)	(0.001)
gov	−0.012	−0.001	−0.005	−0.007	−0.001	0.015 *
	(0.876)	(0.982)	(0.578)	(0.569)	(0.974)	(0.093)
Constant	0.277	−5.023 ***	0.874 ***	0.191	−1.125 **	0.835 ***
	(0.328)	(0.002)	(0.000)	(0.332)	(0.037)	(0.000)
Observations	1.898	1.889	1.980	1.925	1.906	2.004
City FE	YES	YES	YES	YES	YES	YES
Time FE	YES	YES	YES	YES	YES	YES
AR(2)	1.455	−1.131	0.902	1.491	1.188	0.928
ar2p	[0.146]	[0.258]	[0.367]	[0.136]	[0.235]	[0.353]
Hansen test	23.685	202.741	2.453	14.519	116.950	28.279
hansenp	[0.128]	[0.375]	[0.293]	[0.338]	[0.903]	[0.450]
Number of cities	269	268	270	269	268	270
(**b**)						
	**(7)**	**(8)**	**(9)**	**(10)**	**(11)**	**(12)**
**MEDIATORS**	**Environmental Quality**			**Labor Productivity**		
**VARIABLES**	**GTFP**	**pm25**	**GTFP**	**GTFP**	**Labor**	**GTFP**
L. GTFP	0.660 ***		0.661 ***	0.634 ***		0.315 ***
	(0.000)		(0.000)	(0.000)		(0.003)
pm25	−0.001 **		−0.005 ***			
	(0.041)		(0.000)			
labor				0.003 **		0.004 ***
				(0.017)		(0.007)
persco	0.946 **	131.439 ***		0.802 **	4.392 **	
	(0.022)	(0.000)		(0.047)	(0.041)	
pereco	−0.436 **	−111.041 ***		−0.264 **	−26.657 **	
	(0.035)	(0.000)		(0.044)	(0.010)	
perino	1.355 **	−113.727 **		0.695 **	43.247 **	
	(0.023)	(0.036)		(0.019)	(0.043)	
pergov	−0.970 **	146.621 ***		−1.041 **	−40.247 ***	
	(0.027)	(0.000)		(0.014)	(0.002)	
road	0.002	0.319 ***	0.002 **	0.002 *	−0.078 **	−0.001
	(0.247)	(0.000)	(0.027)	(0.091)	(0.033)	(0.496)
mon	−0.084 **	−6.012 ***	−0.056 ***	−0.046	−6.179 ***	−0.013
	(0.016)	(0.007)	(0.006)	(0.121)	(0.000)	(0.480)
fdi	0.525	300.989 ***	1.040 **	0.100	−23.967	0.103
	(0.189)	(0.000)	(0.033)	(0.737)	(0.408)	(0.761)
pgdp	0.019	−5.691 ***	0.026 **	0.003	4.461 ***	0.024 **
	(0.191)	(0.000)	(0.024)	(0.639)	(0.000)	(0.023)
sec	−0.293 *	41.663 ***	−0.549 *	−0.099	−60.135 ***	−0.136 *
	(0.093)	(0.000)	(0.076)	(0.164)	(0.000)	(0.081)
gov	−0.066	−2.093 *	−0.004	0.020 **	0.436	−0.036 *
	(0.308)	(0.072)	(0.698)	(0.018)	(0.540)	(0.060)
Constant	0.267	−27.809 *	0.793 ***	0.180	51.511 ***	0.703 ***
	(0.303)	(0.065)	(0.000)	(0.459)	(0.000)	(0.000)
Observations	1.926	2.170	2.008	1.907	2.170	2.008
City FE	YES	YES	YES	YES	YES	YES
Time FE	YES	YES	YES	YES	YES	YES
AR(2)	1.453	1.339	1.522	1.521	−1.333	1.277
ar2p	[0.146]	[0.181]	[0.128]	[0.128]	[0.183]	[0.202]
Hansen test	51.691	240.532	4.898	24.585	99.054	2.335
hansenp	[0.102]	[0.112]	[0.298]	[0.266]	[0.241]	[0.311]
Number of cities	269	269	270	269	269	270

Note: Robust and cluster standard errors in parentheses and *p*-value in brackets. *** *p* < 0.01, ** *p* < 0.05, * *p* < 0.1.

**Table 9:** The threshold value of four regime-switching variables and its confidence interval.

Threshold Variable	Dynamic Threshold Model	Threshold Value	Wald Statistics	*p*-Value	Number of Bootstrapping	95% Confidence Interval	
						Lower	Higher
persco	SYS-GMM	0.525	0.027 ***	0.000	1000	0.391	0.598
pereco	SYS-GMM	0.132	0.682 ***	0.000	1000	0.087	0.243
perino	SYS-GMM	0.035	5.204 ***	0.000	1000	0.015	0.085
pergov	SYS-GMM	0.134	2.283 ***	0.000	1000	0.101	0.224

Note: *** indicates significance at the 1% level.

**Table 10:** Estimation results of the dynamic threshold panel model.

		(1)	(2)	(3)	(4)
Threshold Variables		Persco	Pereco	Perino	Pergov
Dependent Variables		GTFP			
L. **GTFP**		0.371 **	0.429 ***	0.338 ***	0.421 ***
		(0.033)	(0.000)	(0.003)	(0.000)
wperexp2×I(tvar < c)		−0.007 ***	−0.006 ***	−0.013 ***	−0.016 ***
		(0.000)	(0.002)	(0.002)	(0.000)
wperexp2×I(tvar ≥ c)		−0.005 **	−0.012 **	−0.006 ***	−0.006 ***
		(0.047)	(0.043)	(0.002)	(0.001)
Turning Point (Yuan)	Below the threshold value	81,000	73,000	41,000	35,000
	Above the threshold value	109,000	38,000	84,000	85,000
Percentage change (%)		34.6%	−48.0%	51.2%	58.8%
wperexp		0.107 ***	0.090 **	0.103 ***	0.109 ***
		(0.000)	(0.015)	(0.001)	(0.000)
road		−0.003 ***	−0.002	−0.003 **	−0.004 **
		(0.008)	(0.157)	(0.031)	(0.011)
mon		−0.020	−0.016	−0.021	−0.016
		(0.122)	(0.209)	(0.109)	(0.174)
fdi		−0.128	0.123	−0.090	0.071
		(0.717)	(0.685)	(0.795)	(0.817)
pgdp		0.000 **	0.000 ***	0.000 ***	0.000 ***
		(0.013)	(0.005)	(0.001)	(0.003)
sec		−0.162 **	−0.144 **	−0.168 **	−0.154 **
		(0.035)	(0.025)	(0.014)	(0.015)
gov		0.001	0.000	0.001	0.000
		(0.821)	(0.961)	(0.927)	(0.972)
Constant		0.669 ***	0.638 ***	0.740 ***	0.616 ***
		(0.000)	(0.000)	(0.000)	(0.000)
Observations		2.008	2.008	2.008	2.008
City FE		YES	YES	YES	YES
Time FE		YES	YES	YES	YES
AR(2)		1.450	1.313	1.173	1.457
		[0.147]	[0.189]	[0.241]	[0.145]
Hansen test		2.203	16.89	9.268	8.181
		[0.332]	[0.531]	[0.507]	[0.225]
Number of cities		270	270	270	270

Note: Robust and cluster standard errors in parentheses and *p*-value in brackets. *** *p* < 0.01 and ** *p* < 0.05.

## Data Availability

The data used in this paper comes from the China Urban Statistical Yearbook, the China Urban Construction Statistical Yearbook, the EPS database, economic and social development reports of each city, and the statistical yearbooks published on the official websites of each city’s statistical bureaus.
